# HandKAchip - Hands-free killing assay on a chip

**DOI:** 10.1038/srep35862

**Published:** 2016-10-24

**Authors:** Kyung Suk Lee, Lucy E. Lee, Erel Levine

**Affiliations:** 1 Department of Physics and Center for Systems Biology, Harvard University, Cambridge, MA 02138, USA.

## Abstract

Small animals such as the roundworm *C. elegans* are excellent models for studying bacterial infection and host response, as well as for genetic and chemical screens. A key methodology is the killing assay, in which the number of surviving animals is tracked as a function of the time post infection. This is a labor-intensive procedure, prone to human error and subjective choices, and often involves undesired perturbation to the animals and their environment. In addition, the survival of animals is just one aspect of a multi-dimensional complex biological process. Here we report a microfluidic-based approach for performing killing assays in worms, compatible with standard assays performed on solid media. In addition to providing accurate and reproducible survival curves at a considerably reduced labor, this approach allows acquisition of a multitude of quantitative data with minimal undesired perturbations. These measurements are obtained automatically at a worm-by-worm resolution using a custom image processing workflow. The proposed approach is simple, scalable, and extendable, and is significantly more economical than standard manual protocols.

With virtually all organisms constantly exposed to a multitude of microbes, it is important to develop a fundamental understanding of how bacterial infection develops to pathogenesis and how animals interact with the pathogens and defend against them. *Caenorhabditis elegans* is a widely used model host for bacterial infection^1^. *C. elegans* is a free-living nematode that feeds on bacteria. It is a relatively simple and genetically tractable host^2^, and its transparent body is well suited for visualization of development and infection processes and for *in vivo* monitoring of genes induced by infection. Since the innate immunity of *C. elegans* involves evolutionarily conserved molecular pathways shared with mammalian hosts^1^,^3^, certain aspects of mammalian pathogenesis can be examined using this model animal; see refs 4, 5, 6, 7, 8 for recent reviews.

The killing assay is one of the standard methods for studying pathogenesis and host defense. A standard killing assay is performed on an agar plate with a lawn of pathogenic bacteria, following a specific procedure^9^. The number of live, dead, and lost worms is counted until no viable worm is left on a plate to obtain the *survival curve* (time course of the survival probability), a measure of pathogen susceptibility. Killing assays serve as a powerful tool for elucidating mechanisms of pathogenesis and immune response, as well as for genetic and chemical screens. Using these approaches, several evolutionarily conserved signaling pathways involved in host immune response have been characterized^3^,^10^,^11^. Of particular importance is the p38 MAPK pathway^12^,^13^,^14^,^15^,^16^, involved in activation of immune response to multiple pathogens^17^,^18^,^19^,^20^. Other pathways involved in host defense include the insulin-signaling pathway^21^,^22^,^23^,^24^, the TGF-β pathway^25^ and more. A myriad of factors, including age^26^,^27^, stress response^28^,^29^,^30^,^31^, and the nervous system^32^,^33^, were shown to affect immune functions in the worm. Still, despite their proven usefulness it should be noted that survival curves capture just one aspect of pathogenesis, a complex and dynamic biological process occurring at the interface of two different species.

Performing a killing assay has several impediments. Manual counting is prone to human error and perturbs the worm population. To score the survival of worms, plates are repeatedly opened and hence dried during every measurement. Motionless worms, which become prevalent as the bacterial infection progresses, requires touching their noses to determine whether they are dead or alive^9^. This could take longer than a few minutes depending on the number of worms on a plate and the skill level of an experimentalist, making the assay less reproducible. Besides the mechanical perturbation, plates are dislocated from the incubator and exposed to a different temperature, unless incubation and measurement are performed in a temperature controlled room.

A measurable portion of worms on an agar plate is lost during a killing assay. The lost worms, which were not killed by pathogen, should be censored to produce accurate survival curves. Although the number of the lost worms is not overwhelming, it is still significant enough to necessitate censoring and unbiased correction methods such as Kaplan-Meier estimator^34^. Failing to identify all dead worms would overestimate the survival probability. However, finding all dead worms is not a trivial task because dead worms are degraded in several hours leaving only faint traces of their cuticles^4^,^35^. This is again subject to the skill level of an experimentalist who is required to count every 6–8 hours over several days.

Finally, pathogenesis is confounded by the foraging behaviors of worms, as they spend different amount of time dwelling on the bacterial lawn and roaming on and off it^36^,^37^,^38^. As the survival of worms depends on pathogen uptake, different foraging behaviors could have an impact on the killing dynamics: for example, a polymorphism in *npr-1*, which encodes a homolog of the mammalian neuropeptide Y receptor, demonstrates increased pathogen susceptibility because worms carrying some alleles of this gene do not leave the lawn^32^. To decouple foraging behavior from pathogenesis of worms, it is possible to cover the entire surface of the plate with bacteria; this however leads to a significant increase in the loss of worms, as worms are pushed to crawl up the plate edges.

In the last decade, multiple reports have demonstrated benefits of microfluidic tools in studying worms^39^,^40^,^41^. The air-permeable material polydimethylsiloxane (PDMS)^42^,^43^ is suitable for handling live animals. Performing experiments on a small chip rather than on a 6 cm plate allows imaging at a better spatial resolution, which usually comes with a higher time resolution. Imaging worms on a chip often provides superior imaging quality to agar plates or agar pads and suffers much less background noise. Also, custom designed capillaries and chambers provide unique opportunities to manipulate worms confined in a microfluidic device for the purpose of the experiment.

Here we report a Hands-free Killing Assay on a microfluidic chip (HandKAchip), which not only addresses the issues of the standard killing assay on an agar plate, but also provides multitudes of quantitative data as well as added control capabilities. HandKAchip automates the measurements and therefore minimizes manual manipulation and collateral perturbations, and achieves much better time resolution than the standard killing assay. Since worms are confined in a microfluidic chamber, worms do not escape during the assay, eliminating the necessity of censoring and unbiased estimators. Rapid changes to the environment, such as removal of the pathogen, are made possible with negligible perturbation to worms and minimal risk of contamination. Importantly, for better understanding the progression of pathogenesis, various additional measurements can be made simultaneously with the killing assay. Here we demonstrate, for example, the measurement of pathogen accumulation, gene expression, metabolic fitness, and motility. Quantitative measures are obtained at a worm-by-worm resolution using a robust algorithm for identifying single animals even in cases when they are clustered together. Our experimental approach presents a unique opportunity to study complex and dynamical biological processes of bacterial infection.

## Results

### Hands-free killing assay on worms confined in a microfluidic chamber

**Figure 1:**
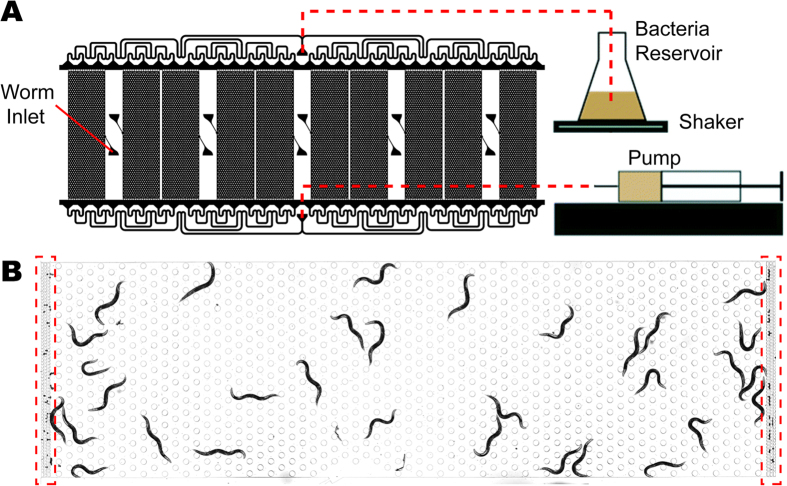
Experimental scheme of hands-free killing assay on a chip. (**A**) Worms are loaded into chambers via worm inlets. Bacterial suspension is drawn into the device from the inlet (top, red dashed line) by a mechanical pump connected to the outlet (bottom, red dashed line). The device is symmetric, so inlets and outlets are interchangeable. The bacterial reservoir is constantly homogenized on a shaker. (**B**) A bright-field image of worms in a single chamber. A hexagonal array of micro-pillars provides structural stability and supports worm motility. Worm-filters (marked by red dashed boxes) prevent adult worms from escaping while allowing progenies and liquid to pass through.

The HandKAchip experimental scheme is depicted in Fig. 1. Worms are loaded into a microfluidic chamber and kept in a temperature controlled room (25 °C) with no further manual perturbations. The entire chip is automatically imaged every hour for the duration of the killing assay (~4 days). The worm-filters located at the top and bottom ends of each chamber forbid worms from leaving the chamber, while allowing progenies and bacteria to pass through (Fig. 1B). Constant flow of pathogen suspension from a reservoir on a shaker goes through the chip at the rate of 10 μL/min and is distributed evenly among 10 chambers via the tree-like channel structure (Fig. S1). This steady replenishment of the liquid in a chip ensures a constant environment for the worms throughout the assay. The concentration of bacterial suspension is uniform all through the chamber, thus eliminating the influence of worm foraging on and off the pathogen. The chamber consists of hexagonal array of micro-pillars as in the artificial soil device^44^,^45^, to provide an environment where worms can crawl as they would on an agar plate or in the soil.

Worms in the chamber exhibit a superb contrast to the background (Fig. 1B), enabling automatic detection (Materials and Methods). On the other hand, on a plate the bacterial lawns of certain pathogens such as *P. aeruginosa* can be very thick and opaque, which yields poor contrast between worms and the background.

**Figure 2:**
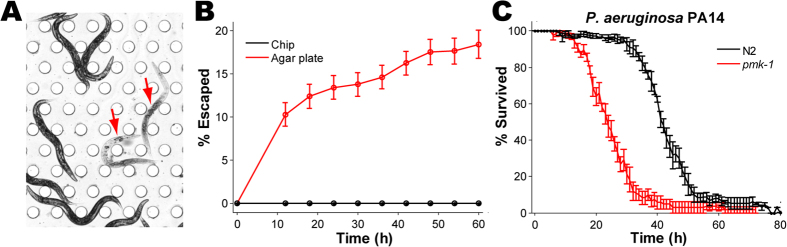
Killing assay on a chip. (**A**) A bright-field image of wild-type worms exposed to *P. aeruginosa* strain PA14, 32 hours post infection, illustrating the contrast between dead worms (arrows) and live ones. (**B**) The time courses of the percentage of escaped worms exposed to PA14 on an agar plate (red), and loaded on a chip with *E. coli* OP50 (black), respectively. (**C**) Survival curves of wild-type worms (N2, black) and *pmk-1* null mutants (red), exposed to PA14 on a chip. Error bars are standard errors in both (**B**,**C**).

To automatically measure the survival curve we define a robust criterion for identifying worms as dead or alive. In our approach, a worm is counted dead when its opacity in a bright-field image drops below a fixed threshold (Fig. 2A), taking advantage of the poor contrast of a degrading dead worm to the background^4^,^35^. This method is not sensitive to the choice of threshold as the opacity of a dead worm drops rapidly, revealing dramatic difference between dead and alive in 4 hours (Movie S2). Thus different choices of threshold shift the estimated time-of-death by no more than 2 hours, significantly less than the time resolution of a standard killing assay (6–8 hours^9^,^12^).

A significant advantage of killing assay on a chip comes already from the fact that worms cannot escape the chamber. Figure 2B compares the time course of the fraction of lost worms on a chip and on a standard killing-assay plate. While 10 to 20% of worms were lost over the course of a standard killing assay necessitating censoring and the Kaplan-Meier estimator, no worm was lost on a chip. This increases the statistical strength and confidence for a given number of worms. It also simplifies the calculation of survival curves and suppresses the influence of human errors in identification of all dead worms.

### Hands-free killing assay on a chip is compatible with the standard killing assay on a plate

As a proof of principle, we measured the survival of worms fed on *Pseudomonas aeruginosa*, one of the best-studied bacterial pathogens of *C. elegans*. This ubiquitous Gram-negative bacterium is found in aquatic environments as well as in the soil. It is an important opportunistic human pathogen that causes infection in immunocompromised and cystic fibrosis patients^46^. Particular strains of *P. aeruginosa*, such as the clinical isolate PA14, induce a lethal intestinal infection in *C. elegans* requiring virulence factors also involved in infection of mammalian hosts. Infected worms exhibit gradual declines in motility, pumping, and egg laying, before they finally die prematurely.

Wild-type animals and *pmk-1* deletion mutants were exposed to PA14 in SK medium (OD_600_ = 4). *pmk-1* encodes for p38 MAPK which functions in an evolutionarily conserved innate immunity pathway, and *pmk-1* mutants exhibit significantly increased pathogen susceptibility^12^,^17^,^47^,^48^. The survival curves obtained automatically from the chip assay (Fig. 2C) were comparable to those obtained in a standard killing assay (Fig. S2A). Importantly, *pmk-1* animals show increased susceptibility to the pathogen. This demonstrates that killing assay on a chip is compatible with killing assay on a plate, and not with hypoxia-induced killing by PA14 in a liquid culture^49^. As a control, worms fed by benign *E. coli* OP50 in S-medium (OD_600_ = 4) survived well over the 3 days of the experiment (Fig. S2B).

Using the same approach we assayed the survival of worms on the less virulent strain *P. aeruginosa* PAO1. The survival curve (Fig. S2C) demonstrate clearly that worms are less susceptible to this strain than to the clinical isolate PA14. On the other hand, the significant decline in worm survival on this pathogen, as compared with benign *E. coli*, is evident already on day 4.

### Exchanging the media in a chip leads to negligible perturbation and minimal contamination

Recovery from infection due to removal of the pathogen, introduction of drugs, or changes in other environmental factors, can be instrumental in understanding the dynamics and causes of infection. For example, removal of PA14 in the first 12 hours, but not later, leads to full recovery^9^. In a standard killing assay, it is possible to change the environment by moving worms from plate to plate. To minimize transfer of environmental factors from one plate to the next, worms are washed repeatedly before the transfer. This procedure suffers two major shortcomings. First, it introduces a significant perturbation, as a worm in liquid exhibits a thrashing behavior that involves a distinct gene activity, including AMP activated protein kinases^50^,^51^. Second, even after excessive washing worms carry environmental factors in their intestine, whose content is defecated on the new plate. In particular, bacteria carried in the worm intestine can grow on the new plate and compete with existing bacteria (see, *e.g.*, Fig. S3).

**Figure 3:**
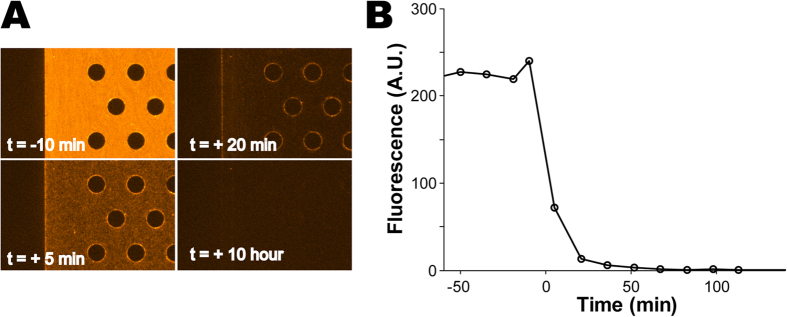
Exchanging the media on a chip. (**A**) Fluorescence images of a part of one chamber, taken at different times. A culture of *P. aeruginosa* PA14 bacteria constitutively expressing RFP was switched at time *t* = 0 to media with dark *E. coli* OP50. For reference, the darker area on the left of all images is a PDMS wall, inaccessible to bacterial suspension. (**B**) The time course of mean fluorescence from the chip.

In contrast, the microfluidic environment can be exchanged with no additional perturbation to the worms, and the continuous replenishment of media and food limit the cross-contamination effect of intestine-borne bacteria. To show this, we fed worms for 6 hours with PA14 bacteria that constitutively expressed red fluorescence protein (RFP), before switching the media with non-fluorescent OP50. Figure 3A shows red fluorescence images from a chamber populated by 30 worms at different times before and after the exchange. The rapid decrease in the fluorescence from the chamber (Fig. 3B) indicates that PA14 was quickly and efficiently replaced with OP50. Defecated bacteria are continuously removed and are never allowed to grow in the chamber.

### Simultaneous measurements of multiple aspects of bacterial infection

Premature death is the final outcome of intestinal bacterial infection in worms, but the decline in survival is just one aspect of this complex and dynamic process. Pathogens populate the intestine of worms, while the hosts execute genetic programs to defend themselves from the pathogen. As pathogenesis progresses, various aspects of the fitness of worms (associated with healthspan parameters^52^) deteriorate over time. A microfluidic-based approach offers an opportunity to study the multifaceted biological process of pathogenesis, by combining multiple simultaneous quantitative measurements.

**Figure 4:**
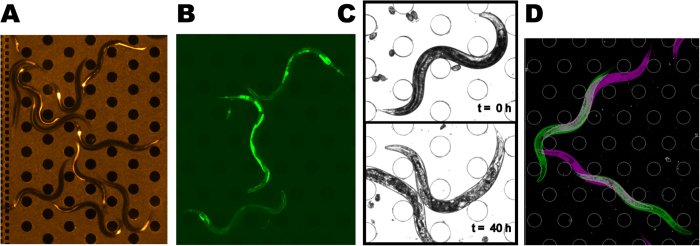
Simultaneous measurement of different aspects of infection. (**A**) **Bacterial load in the intestine**: wild-type worms fed by *P. aeruginosa* PA14 bacteria constitutively expressing RFP, 6 hours post infection. Fluorescence inside the body of worms comes from bacteria in the intestine. (**B**) **Gene expression**: transgenic worms carrying a transcriptional reporter of irg-1 (*irg-1::GFP*), 12 hours after exposure to PA14. (**C**) **Body color**: bright field images of worms immediately before and 40 hours after exposure to PA14. (**D**) **Motility**: Superposition of two bright-field images of the same area, taken 1.3 sec apart (magenta: earlier, green: later).

Increased susceptibility due to mutations or environmental factors can be the result of reduced tolerance or reduced resistance^53^. To discriminate the two, it is useful to measure the load of bacteria in the infection site. For intestinal infection of the worm, this can be done by tagging bacteria with a fluorescent marker, taking advantage of the transparency of the worm. In Fig. 4A, for example, we image a killing assay on a chip with a strain of PA14 constitutively expressing RFP. Six hours post infection, the intestine of most of worms is filled with the pathogen.

Many genes exhibit dynamical transcriptional response to infection. Among these, *irg-1* (*infection response gene 1*) is specifically and strongly induced by *P. aeruginosa* strain PA14, making it a convenient tool for probing early responses to *P. aeruginosa* infection^17^,^54^. The pattern of changes in gene expression can be monitored during the killing assay using fluorescent reporters. For example, Fig. 4B shows transgenic worms expressing *irg-1*::*GFP* 12 hours post infection by PA14. Notably, the survival kinetics of the reporter strain was comparable with those of wild type animals in the presence of either pathogen (Fig. S4A) or benign food (Fig. S4B).

The impact of infection on the health of the animal can be observed long before it is killed. For example, as a proxy to the metabolic impact of infection, one can use the microfluidic setup to follow the decline in worm opacity^55^. Well-fed worms appear opaque in bright-field images due to dark gut granules, presumed to be the major sites of fat stores in *C. elegans*^56^, while starvation leads to a brighter body color^57^. Similar to worms undergoing starvation, worms exposed to PA14 become increasingly pale over time (Fig. 4C). Finally, decline in motility is a known symptom of PA14 infection in worms^9^. The dynamics of this decline can readily be observed in the microfluidic environment. The decline in motility can be observed in the microfluidic chamber by taking 2 images separated by about 1 second, and measuring the displacement of each worm (Fig. 4D).

### Quantitative measurements at a worm-by-worm resolution

The high quality time lapse imaging from the microfluidic chambers allows quantification of the dynamics of different aspects of infection. For fluorescent reporters and body color measurements, the simplest way to obtain quantitative data is to identify all pixels associated with worms in each frame, and average their intensity. In particular, there is no need to associate each pixel with one worm or another.

However, the population average may be telling only part of the story, especially in the presence of high worm-to-worm variability, or in cases where the population of worms becomes inhomogeneous. Estimating higher order statistics necessitates identification of individual worms in the frame, which is challenging whenever worms in the chamber are not well separated. In addition, some aspects – such as worm motility – can only be measured in individual animals. While continuous imaging of individual worms has been achieved by segregating the animals into separated compartments^58^,^59^,^60^ or by longitudinal tracking^61^,^62^, neither is ideal for a killing assay that requires monitoring a large population of worms for hours and days. Instead, we developed an algorithm that identifies individual worms in a crowded population.

**Figure 5:**
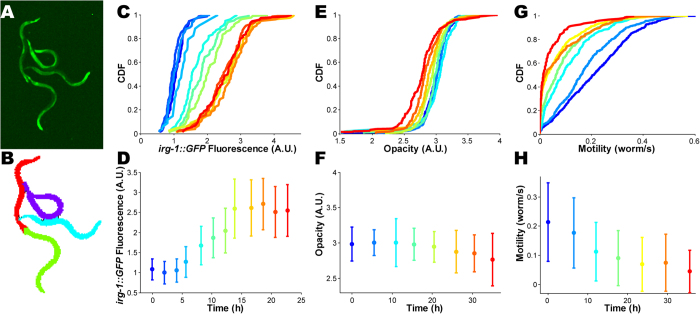
Single-worm statistics of quantitative measurement. (**A**) Four worms in contact with each other (*irg-1::GFP* reporter strain, fluorescence image). (**B**) The cluster of worms in panel A was separated into individual worms using an automated image analysis workflow, described in the text. (**C**,**E**,**G**) The time courses of cumulative distribution function (CDF) of *irg-1::GFP*, opacity, and motility of single worms, respectively. The measurement time is color-coded as in bottom panels. Worms were exposed to *P. aeruginosa* PA14 at time *t* = 0. (**D**,**F**,**H**) The time courses of the mean (circles) and standard deviation (bars) of *irg-1::GFP* fluorescence, opacity, and motility of single worms, respectively.

To extract the fluorescence or the body color of individual worms from the acquired images, it is necessary to identify the pixels that correspond to each worm. The high contrast between the worms and the background in the microfluidic device allows identification of worm-associated pixels and unambiguous definition of connected components of these pixels, each representing one or more worms (Fig. 5A, Materials and Methods). Components that contain multiple worms (Fig. S6A) need to be separated into single-worm components (Fig. 5B). In general, it is a very difficult problem, which is significantly simplified by the fact that the height of the chambers of the device does not allow worms to cross on top of each other. This constraint, along with the observation that a sharp turn in a component boundary (green pixels, Fig. S6C) indicates a contact point between two worms, make the separation problem solvable in most cases. Using these features, we developed a robust algorithm for separating multi-worm components to single-worm ones (Fig. S6, Materials and Methods).

Identifying individual worms makes it possible to obtain the quantitative measurements at a worm-by-worm resolution. In Fig. 5 we demonstrate this capability by plotting, for 3 aspects of infection, the cumulative distribution functions (CDF) over the population of worms, as well as the mean and variance. Figure 5C,D show the dynamics of induction of *irg-1* expression, quantified as the total fluorescence per worm. As previously reported, the expression of *irg-1* increases with time in worms fed by PA14^17^,^54^ (Fig. 5C, Movie S3), but not in worms fed by benign bacteria (Fig. S5A,B). Interestingly, the distribution of *irg-1* expression significantly broadens in time.

The decline in body opacity during infection is quantified in Fig. 5E,F, and is contrasted with the increase in opacity in well-fed worms (Fig. S5C,D). Notably, the statistical significance of this decline is only appreciated when using the entire distribution function (p-value < 10^−5^ for all non-consecutive curves pairwise, Kolmogorov–Smirnov test, Fig. 5E), but it would not have been if only population averages were available.

Finally, motility of the worms was quantified by comparing two images, taken ~1 sec apart, and counting the fraction of pixels covered by each worm in the later image but not in the earlier. Figure 5G,H show the decline in motility over time. Inspecting the CDF, we find that well-fed worms exhibit a wide range of speeds, from perfect stillness to fast motion, and that the decline in motility of infected worms occurs predominantly via a decrease in the maximal observed speed, reminiscent of what is observed in aging worms^63^.

### Scalable Design

In all the experiments presented here we used 20-chamber PDMS chips mounted on standard 2′′ by 3′′ glass slides. Increasing throughput can easily be done by adding more chambers on a larger slide. A standard mounting frame from the manufacturer (without modification) can easily fit slides for 120-chamber chips. In our experimental setup (Materials and Methods), data acquisition typically takes less than 5 seconds per chamber, such that even a 120-chamber chip would permit imaging at a 5-minute resolution, well beyond what is required by the typical time scale of infection dynamics.

## Discussion

Measuring the survival kinetics of the simple model organism *C. elegans* in the presence of pathogenic bacteria and fungi have been useful for understanding mechanisms of microbial infection and host immune responses. The physically demanding schedule of the assay, along with several technical drawbacks, have been calling for automating the killing assay. Here, we present hands-free killing assay on a microfluidic chip. Supplemented with an efficient algorithm that takes advantage of the properties of the chip to identify individual worms in a crowded population, our approach successfully automates the experiment and minimizes manual perturbation, while enabling simultaneous quantitative measurements of other aspects of pathogenesis.

We demonstrated the strengths of this approach by characterizing the intestinal infection of *C. elegans* by a clinical isolate of the opportunistic pathogen *P. aeruginosa*. Under standard conditions, we illustrated how survival, motility, gene expression, and bacterial load can be measured during the entire infection process, until all worms succumb to the infection after 3 days. Importantly, the device remains intact throughout the experiment, and can therefore be used in the same way for studying infection by other pathogens, such as *Salmonella typhimurium* or *Enterococcus faecalis*, where some worms survive for 5 or 6 days.

Several applications require rapid, and even repeated, change of the environment in which a killing assay is performed. For example, as described above, testing the persistence of an infectious agent requires rapid removal of the agent from the environment. As another example, in chemical screens for compounds that combat infection it is useful to introduce a chemical compound into the environment in a short pulse, whose timing and duration need to be optimized. Further, to test the effect of cross-interaction between bacterial species, one may be interested in exposing animals to different microbial species in a specific temporal order.

When performed on plates, changes in the environment involve transfer of animals between plates. To minimize cross-contamination, the transfer step involves multiple rounds of washing, as well as transient incubation on empty plates. This procedure constitutes a major perturbation to the animals, including hypoxia and starvation, thus complicating the interpretation of subsequent results. Moreover, even with a careful transfer, cross-contamination is inevitable, and in some conditions can obscure the results and lead to wrong interpretation. In contrast, performing the assay in a microfluidic device permits a continuous exchange of the environment, facilitating rapid and reversible transfer from one environmental condition to another, complete clearance of contaminants, and discrimination between transient and truly persistent infection. These capabilities are unique to a microfluidic-based approach.

Many questions that focus on life histories of individual worms, as well as learning and behavior, require longitudinal tracking of individual worms. Several available approaches to automated imaging and image analysis allow tracking, but typically require imaging of isolated worms or worms at very low density^64^,^65^,^66^. Implementing longitudinal tracking in the HandKAChip device could potentially bypass this requirement, provided that images are taken at high frequency and that the image processing software is further developed to maintain the identities of individual animals across frames.

Much concern has been raised recently about the reproducibility of published results in the biomedical literature^67^,^68^. An important potential source is human error, especially in cases that rely on a judgment call made by an experimentalist, based on some ill-defined criteria. In the context of killing assays, two such calls are the identification of worms as dead or alive, and the censoring of missing worms. The level of accuracy of these calls is influenced by the experience and training of the person performing the experiment, but also by the fact that the experiment is performed around the clock for several days. The approach proposed here, which automates the essay, prevents the need for censoring, and classifies worms based on robust quantitative criteria, is expected to make the results of the essay more reliable and reproducible.

The advance in microfluidics and image processing techniques offers a unique opportunity for obtaining more accurate, reproducible and undemanding survival curves, as well as a multitude of quantitative data with minimal undesired perturbations. The compact design of our chip and the short measurement time allows scaling up the experiments for higher throughput. Moreover, the simplicity of the design should make it broadly accessible and useable. Precise layout of the device, suggested part list, and a MATLAB implementation of the image analysis algorithm is available at https://github.com/eerunid/HandKaChip.

## Materials and Methods

### Worm Strains

All strains were maintained on standard nematode growth medium (NGM) plates seeded with *E. coli* strain OP50^69^ at 15 °C. Adult hermaphrodites of the following strains were used: N2 Bristol strain, KU25 *pmk-1(km25)IV*, AU0133 *agIs17(irg-1::GFP)* obtained from Ausubel lab^70^.

### Bacterial Strains

*Pseudomonas aeruginosa* strain PA14 was used in all assays, except one experiment where *E. coli* strain OP50 was used (Fig. 3). To visualize bacterial accumulation, PA14 with constitutive DsRed.T3 (DNT) expression (PA14 pAA100 obtained from Ausubel lab^71^) was used.

### Killing assay

For a killing assay on a plate, we followed the standard protocols from the literature^9^,^70^. Briefly, an overnight culture of PA14 grown in LB at 37 °C was spread on a SK-agar plate and then incubated at 37 °C for 24 hours. The plates were moved to 25 °C and incubated another 24 hours. Age-synchronized adults were prepared by hypochlorite treatment and L1 arrest. Synchronized L1 larvae were plated on a standard NGM-OP50 plate and cultivated at 25 °C for 48 hours. The plates were washed with M9 to propagate worms to PA14 SK-agar plates, and the plates were incubated at 25 °C after completely drying the excessive M9 on the plates. Worms were counted as dead when they failed to respond to nose-touch by a worm pick. Lost worms or worms found dead on the plate wall were censored.

For killing assay on a chip, an overnight culture of PA14 was centrifuged and resuspended to OD_600_ = 4 in SK-medium. The bacterial suspension was incubated at 37 °C for 24 hours and at 25 °C for another 24 hours while shaking. Synchronized adults in M9 were loaded onto the microfluidic device. After removing bubbles, the media was switched to PA14 in SK-medium to start the assay. The bacterial suspension was filtered through a 5 μm syringe filter to remove big aggregates, which could block the flow in the microfluidic device and injected into the device at a rate of 10 μL/min. The assays were usually stopped when all or almost all worms were killed (~80 hours), but could be extended as long as desired, except in rare cases where biofilm was formed in the chamber. Removal of aggregates and replacing clogged filters helped to suppress biofilm formation.

The chip was mounted on a compound microscope with a motorized stage in a temperature controlled room at 25 °C, and were imaged every hour for the duration of the assay. The recorded images were analyzed automatically using custom MATLAB scripts.

### Imaging worms in a microfluidic device

Imaging was done using a Zeiss Axio Observer.Z1 microscope with either EC Plan-Neofluar 2.5x or N-Achroplan 10x objectives, and a Hamamatsu ORCA Flash4.0 camera. For killing assay, transmitted light bright-field imaging without a condenser unit was used. Each channel required 3 and 48 images to cover the entire area with 2.5x and 10x objectives, respectively. 10–20 channels were monitored simultaneously.

### Image analysis

For each image, a mask marking the pixels associated with worms was generated by comparing the intensity of pixels, as worms are much darker than background in bright-field images (and brighter in fluorescence imaging). Each connected component in such a mask corresponds to a worm or multiple worms. With 30–35 worms per chamber, single-worm components are the most abundant. We estimated the mean area covered by a single worm from the distribution of component area, and use this value to estimate the number of worms in each connected component. The average darkness of each single-worm component was compared to a heuristic threshold value (given by the average darkness of its neighborhood plus the standard deviation of the pixel darkness in the image) to differentiate live and dead worms. The precise choice of threshold value is not important due to the dramatic difference in darkness between live and dead animals (Fig. 2A, Movie S2).

To separate a multi-worm component (Fig. S6A) into single worm ones (Fig. S6G), we first identify narrow regions as parts of single worm bodies. These are marked by white lines and a thick colored outlines in Fig. S6B. Thicker regions are left out, because they are likely to be multiple worm junctions. The goal is to connect the worm parts that are disconnected by multi-worm junctions into complete individual worms.

Each multi-worm junction needs to be resolved, such that parts coming from the same worm are connected together. For example, a junction made by two worms (magenta arrow in Fig. S6A) can be resolve in two ways: either by connecting the two parts on the left and the two parts on the right, or by connecting to the two parts at the top and the two at the bottom (as in Fig. S6E). We perform this task by observing the turn-angle along the boundary, and identifying turns that are too sharp to come from a silhouette of a worm (green pixels, Fig. S5C). These connections are prohibited (white markers, Fig. S5D), leaving one possible resolution (Fig. S5E). Cases where the ends of two worms touch each other (green arrow in Fig. S6A) cannot be resolved by turn-angles. Instead, the parts of these worms are connected into one long segment (red in Fig. S5F). Such segments are separated into individual worms using the average length of isolated worms as a ruler (red and green in Fig. S5G).

This algorithm separates worms well most of the time, but it fails if there are too many worms clumped in a single component. The success rate (the number of successfully separated worms as a fraction of the total number of worms tried) was about 80% in our test sets (594 worms separated from 735 worms in multi-worm clusters: 211/260, 150/192, 233/283 in three trials).

## Additional Information

**How to cite this article**: Lee, K. S. *et al*. HandKAchip - Hands-free killing assay on a chip. *Sci. Rep.* **6**, 35862; doi: 10.1038/srep35862 (2016).

## Supplementary Material

Supplementary Movie S1

Supplementary Movie S2

Supplementary Movie S3

Supplementary Information
