# Supplementary material for: HandKAchip - Hands-free killing assay on a chip
Source: Sci Rep. 2016 Oct 24;6:35862. doi: 10.1038/srep35862 (PMC5075874; doi:10.1038/srep35862)
Supplement: Supplementary Information [file srep35862-s4.pdf]

HandKAchip - Hands-free killing assay on a chip

Kyung Suk Lee, Lucy E. Lee and Erel Levine

Supplementary Figures

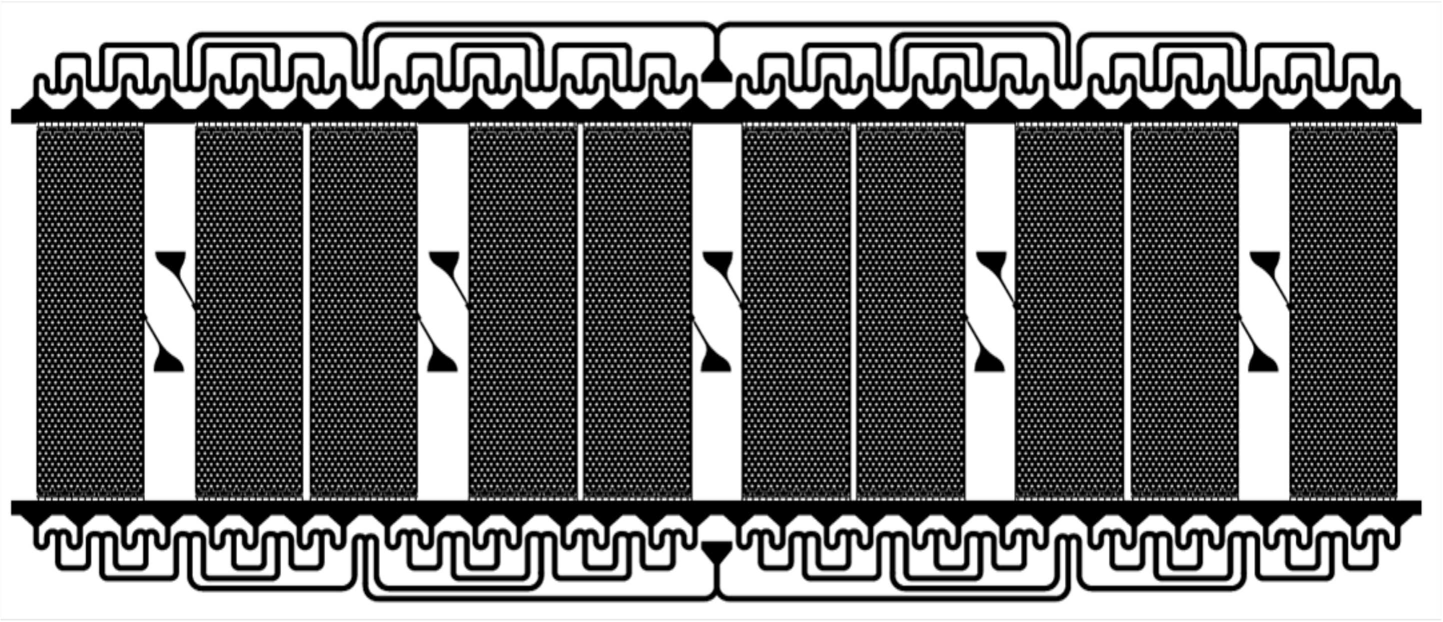

Figure S1. A CAD image of the 10-channel device, related to Figure 1.

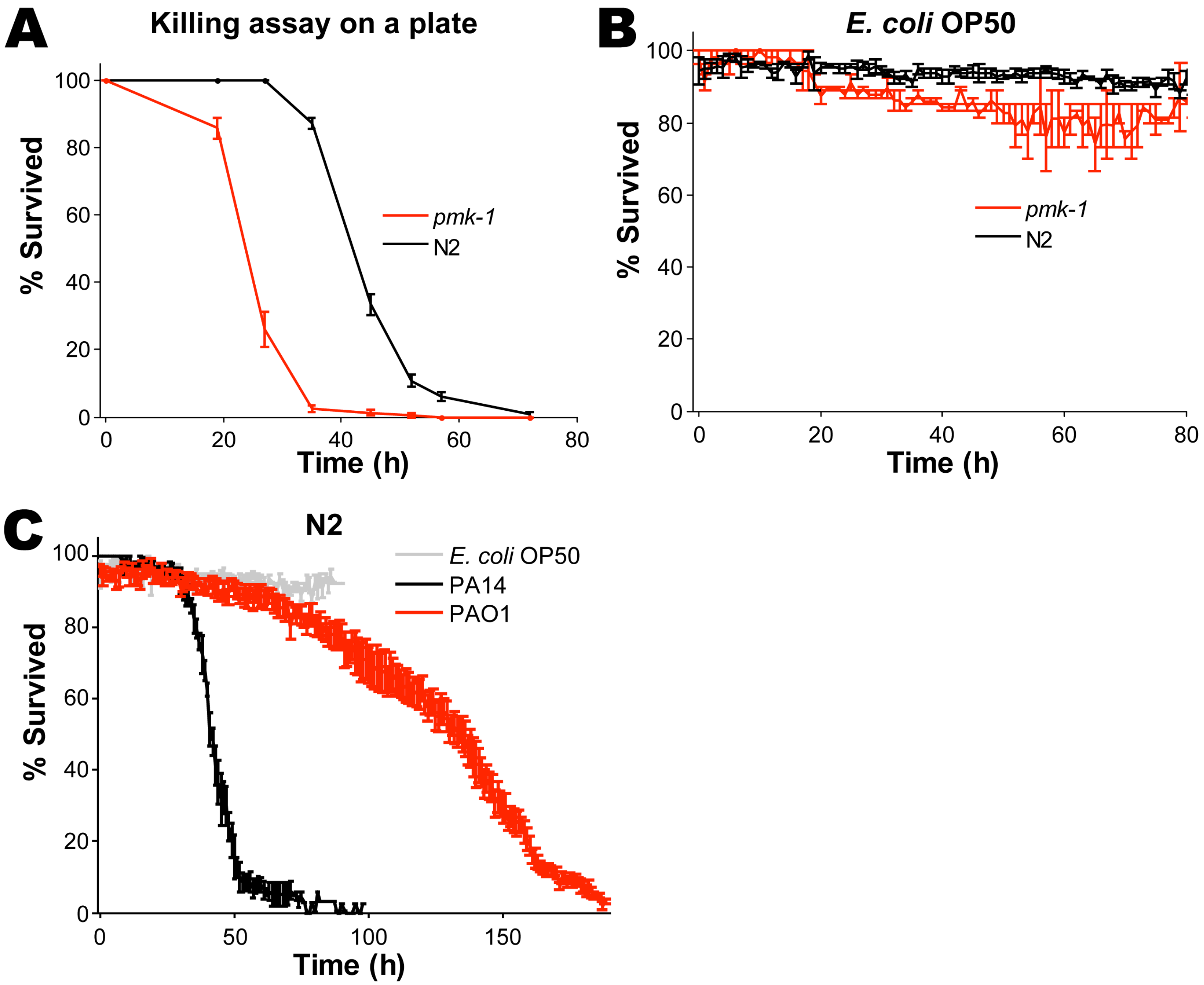

Figure S2. Distinct survival curves on different strains of bacteria, related to Figure 2. (A) The killing curves of wild-type worms and *pmk-1* mutants, measured on a plate. Worms were exposed to *P. aeruginosa* PA14 following the standard killing assay protocol. (B) Survival curves of well-fed wild-type worms and *pmk-1* mutant on a chip (*E. coli* OP50 at OD_{600} = 4). (C) Survival curves of wild-type worms fed with *P. aeruginosa* PAO1 (red), PA14 (black, as in Fig. 2C), and *E. coli* OP50 (gray, as in panel B), measured on chip. Error bars are standard errors.

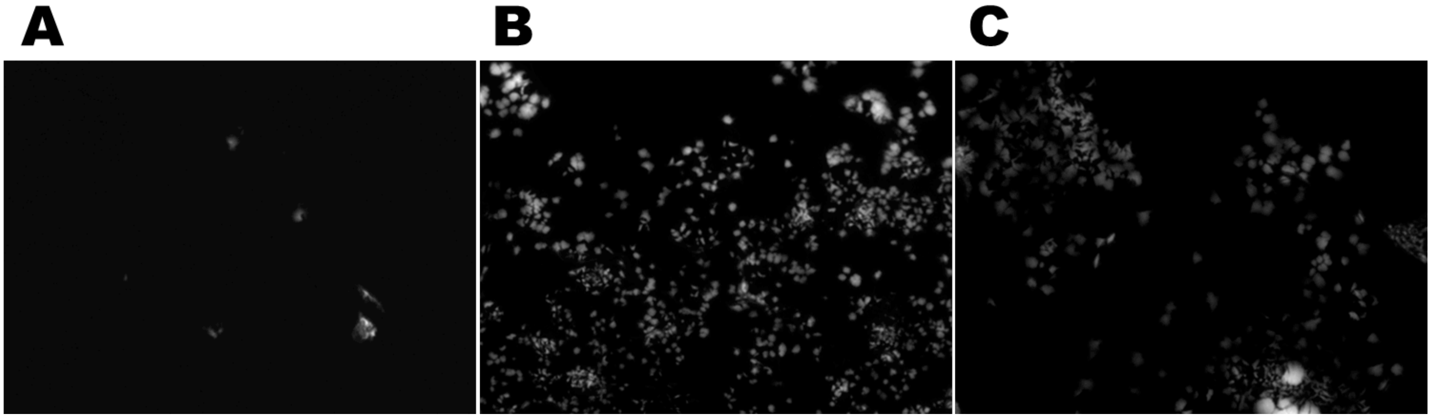

Figure S3. Cross contamination in plate exchange experiments, related to Figure 3. Wild-type worms were picked from a plate covered with *P. aeruginosa* PA14 bacteria constitutively expressing RFP on a plate, washed 3 times, and transferred to fresh plates covered with *E. coli* OP50. Fluorescent images of the latter plate were taken a day later, following an overnight incubation at 37 °C, to reveal PA14 contamination. (A) Worms spent 4 hours on PA14 plates before transfer. (B) Worms spent 12 hours on PA14 RFP. (C) Worms spent 12 hours on PA14 RFP. In this experiment, worms spent 30 minutes on an empty plate after washing and before transfer to the final plate.

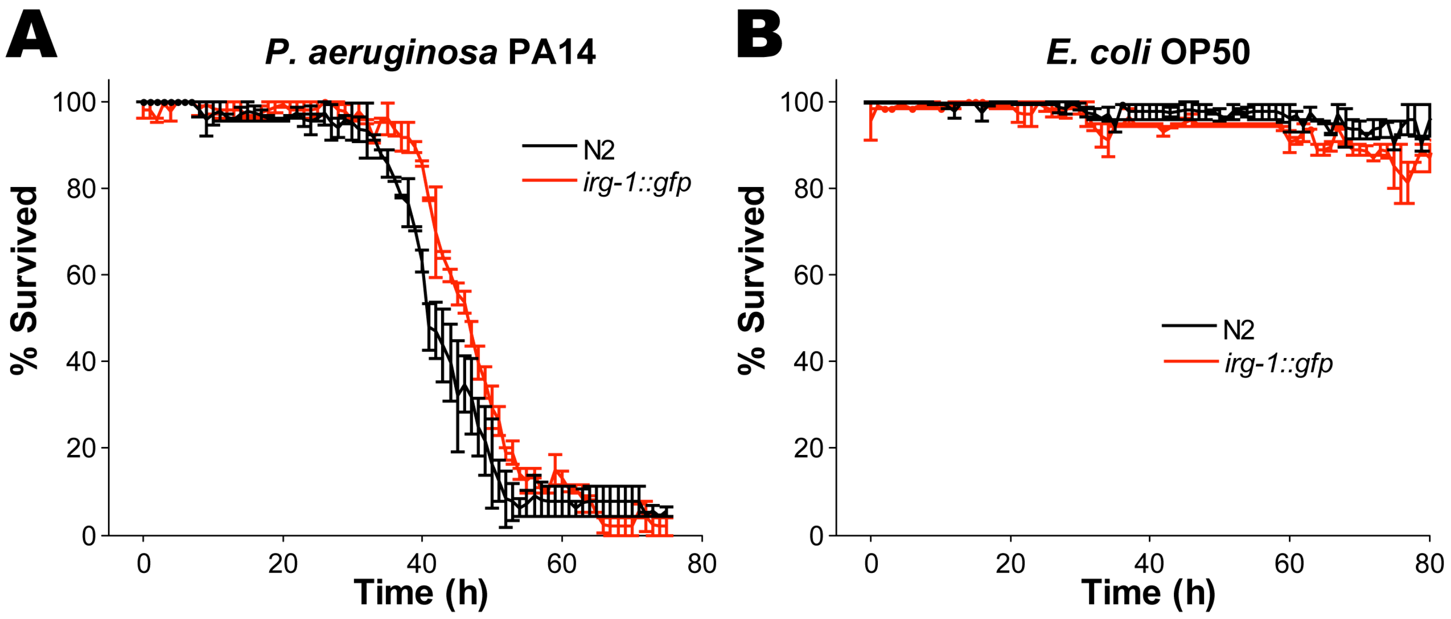

Figure S4. Survival of transgenic worms expressing *irg-1::GFP* is similar to survival of wild-type worms, related to Figure 4. (A) The survival curves of the wild-type and the reporter strain exposed to *P. aeruginosa* PA14. (B) The survival curves of the wild-type and the reporter strain fed by *E. coli* OP50.

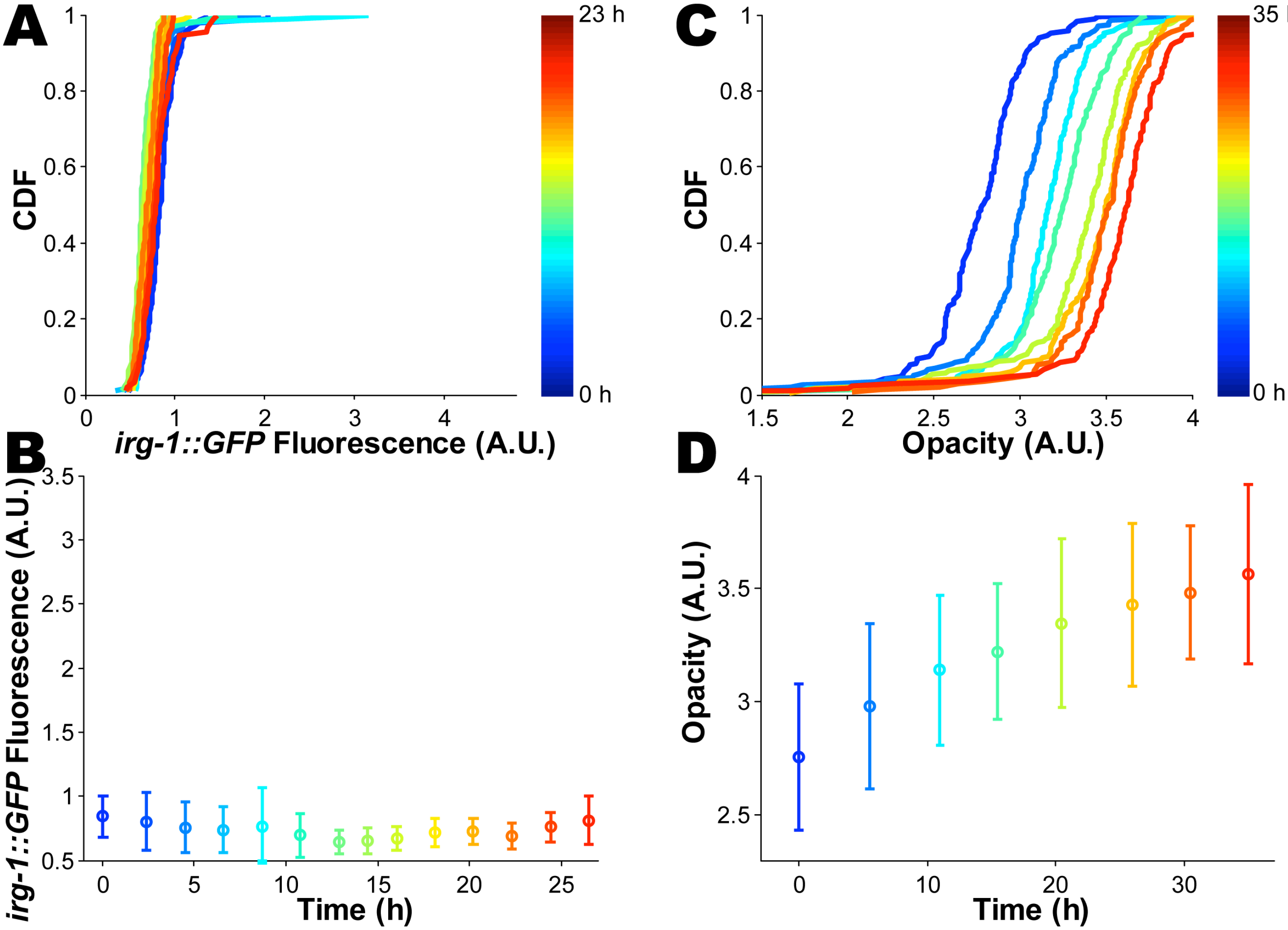

Figure S5. Single-worm statistics of quantitative measurement, related to Figure 5. (A) The time course of CDF of the total fluorescence of well-fed *irg-1::GFP* reporter strain (*E. coli* OP50 at OD_{600} = 4). (B) The mean of the total fluorescence of the worms in (A). (C) The time course of CDF of the opacity of well-fed wild-type worms (*E. coli* OP50 at OD_{600} = 4). (D) The mean of the opacity of the worms in (C). The measurement time in (A,C) is color-coded as in Fig. 5, and the error bars in (B, D) as in Fig. 5.

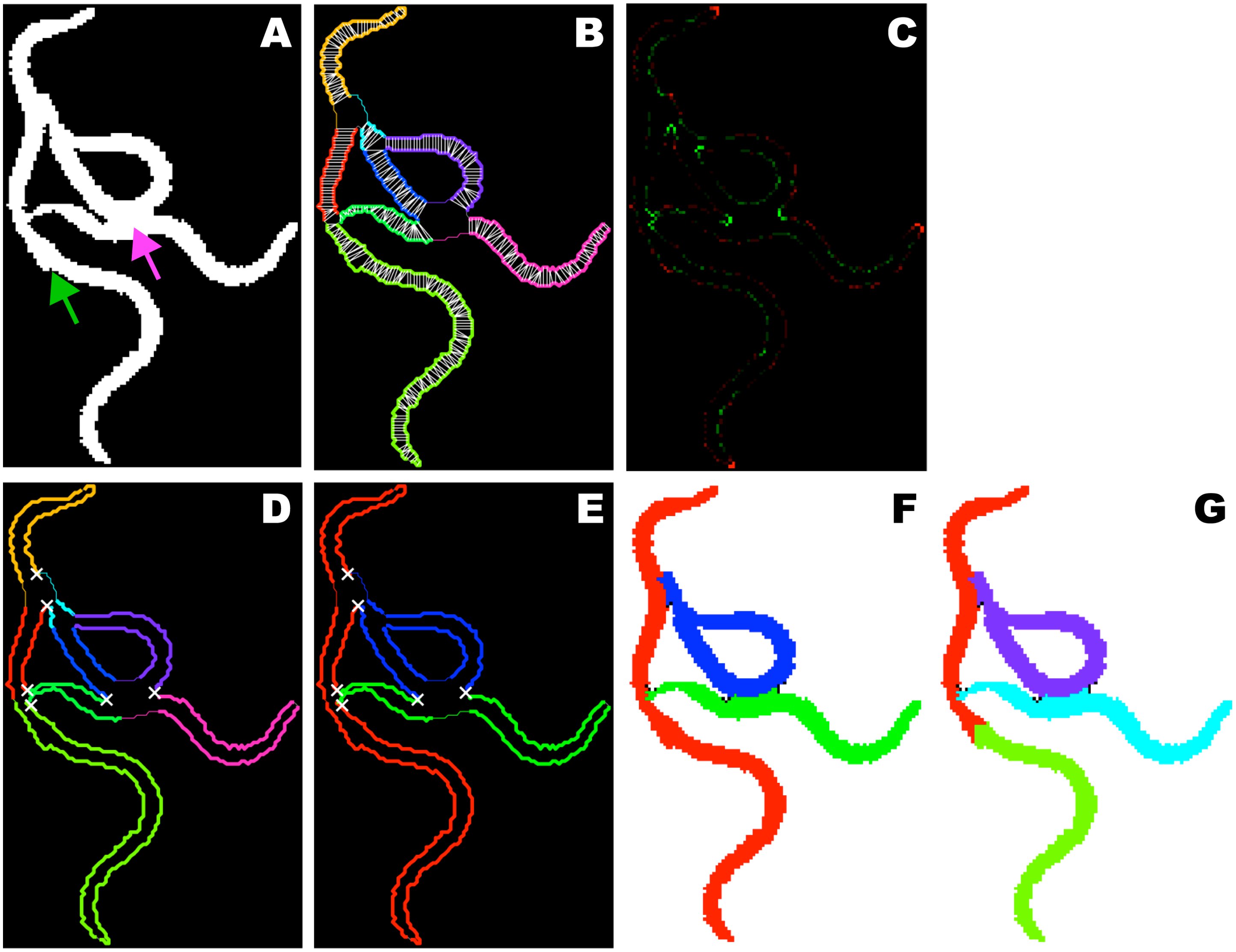

Figure S6. Separating individual worms, related to Figure 5. (A) The connected component related with the worm cluster of Fig. 5A. (B) Regions identified as likely parts of individual worms are marked by thick outlines of different colors. White lines connect left and right sides of the same worm. (C) The turn-angle of the boundary (the difference between tangential vector just before and after each point) is shown color-coded in green and red: green (turning outward from the body), red (turning toward the body). (D) Boundary position identified as contact points between two worms are marked by white symbols. (E) Partial worms are consistently connected into larger parts. (F) Gaps are filled to yield partial separation. (G) Final separation is achieved by cutting long components into individual worms, based on the estimated average length of single worms. Single worms are in different colors.

Supplementary Movie Legends

Movie S1. Time lapse imaging of worms on a chip, related to Figure 1. Wild-type worms exposed to *P. aeruginosa* PA14. Images are taken at a rate of 1 frame per hour, shown at 10 frames per sec.

Movie S2. Time lapse imaging of dying worms on a chip, related to Figure 2. Wild-type worms exposed to *P. aeruginosa* PA14. Images are taken at a rate of 1 frame per hour, shown at 1 frame per sec.

Movie S3. Fluorescence images of *irg-1::GFP* reporter strain, related to Figure 4. *irg-1::GFP* reporter strain were exposed to *P. aeruginosa* PA14. Images were taken at different times (annotated at the top-right corner) before and after the infection.
